# *Elizabethkingia anophelis* Isolated from Patients with Multiple Organ Dysfunction Syndrome and Lower Respiratory Tract Infection: Report of Two Cases and Literature Review

**DOI:** 10.3389/fmicb.2017.00382

**Published:** 2017-03-08

**Authors:** Shaohua Hu, Tao Jiang, Xia Zhang, Yajun Zhou, Zhengjun Yi, Youxi Wang, Sishou Zhao, Mingxi Wang, Desong Ming, Shicheng Chen

**Affiliations:** ^1^Yun Leung Laboratory for Molecular Diagnostics, School of Biomedical Sciences, Huaqiao UniversityXiamen, China; ^2^Department of Medical Laboratory, Institute of Nanomedicine Technology, Weifang Medical UniversityWeifang, China; ^3^Department of Information, Quanzhou First Hospital Affiliated to Fujian Medical UniversityFujian, China; ^4^Department of Clinical Laboratory, Quanzhou First Hospital Affiliated to Fujian Medical UniversityFujian, China; ^5^Department of Microbiology and Molecular Genetics, Michigan State UniversityEast Lansing, MI, USA

**Keywords:** *E. anophelis*, emerging pathogen, multiple organ dysfunction syndrome, lower respiratory tract infection, antibiotics resistance

## Abstract

*Elizabethkingia anophelis*, originally discovered from *Anopheles* mosquito gut, is an emerging pathogen, especially in immunocompromised patients. We isolated two strains of *E. anophelis* from two separate patients with multiple organ dysfunction syndrome and lower respiratory tract infection. In this paper, we reviewed the status of *E. anophelis* infection and its antibiotics resistance from reported cases.

## Background

*Elizabethkingia*, a Gram-negative, aerobic, rod-shaped bacterium, belongs to the family *Flavobacteriaceae* within the phylum *Bacteroidetes*. Four species are assigned to this genus, including *Elizabethkingia meningoseptica, E. anophelis, Elizabethkingia miricola*, and *Elizabethkingia endophytica*, with the first three being considered to be medically important (Kukutla et al., 2013, 2014; Lau et al., 2015; Breurec et al., 2016). *E. anophelis* was initially isolated from the midgut of mosquito *Anopheles gambiae* in 2011 (Kämpfer et al., 2011). Later, it was found in the environment (water and soil) and health care settings (Balm et al., 2013; Teo et al., 2013). It rarely causes infections in healthy people while it is pathogenic to immunocompromised patients with a high mortality rate (Teo et al., 2013; Lau et al., 2016; https://www.cdc.gov/elizabethkingia/outbreaks/). The first reported case of *E. anophelis* infection was meningitis in an 8-day-old girl delivered by cesarean section in the Central African Republic in 2011 (Frank et al., 2013). It also caused nosocomial outbreak in two intensive-care units from a hospital in Singapore in 2012 (Teo et al., 2013). By conventional phenotypic test, an epidemiological survey of bacteremia from five regional hospitals in Hong Kong (from 2004 to 2013) identified 17 cases of *E. anophelis* bacteremia (Lau et al., 2016). *E. anophelis* was implicated in recent outbreaks of infections in the Midwest regions. In Wisconsin, 63 confirmed and 4 possible infections were reported between November 1, 2015 and January 11, 2017. Among these cases, a total of 19 deaths (estimated mortality rate, 28%) were found (https://www.dhs.wisconsin.gov/disease/elizabethkingia.htm). At least 10 *E. anophelis* infections were reported in Illinois (between January 2014 and April 2016) and one in Michigan in March 2016 (https://www.cdc.gov/elizabethkingia/outbreaks/). Most patients were over 65 years old with serious underlying diseases (https://www.cdc.gov/elizabethkingia/outbreaks/). We report here two cases of *E. anophelis* infection at Quanzhou First Hospital, Fujian, China.

## Case presentation

### Case 1

An 82-year-old male patient presenting with lethargy in consciousness, malnutrition, multiple bedsores [an 8 × 10 cm^2^ at level I (containing a 5 × 6 cm^2^ at level II) on the sacrococcygeal region, two 5 × 5 cm^2^ at level I on the both heel, two 2 × 2 cm^2^ at level I on both external malleolus] was admitted on December 14, 2009. He had a history of surgery for esophagus cancer at 73 years old, hypertension at level III, two hospitalizations due to lower respiratory tract infections (treated by azithromycin, cefoperazone/tazobactam, levofloxacin, meropenem, linezolid, piperacillin/tazobactam, and ciprofloxacin) 4 months before this final hospitalization. He demonstrated expectoration and shortness of breath for 7 days before admission. The blood gas analysis and blood biochemical test at admission showed respiratory acidosis (pCO_2_ 97 mm Hg, pO_2_ 122 mm Hg), metabolic alkalosis (pH 7.48, ${\text{HCO}}_{3}^{-}$ > 60 mmol/L, CO_2_ 36.8 mmol/L), very low blood K^+^ (2.1 mmol/L), renal dysfunction (BUN 23.9 mmol/L), and extremely low Cl^−^ (68.6 mmol/L). He came into deep coma with BP 58/34 mmHg within 2 h after admission. Emergency salvage was given, including assisted ventilation, raising BP by dopamine and L-noradrenaline, supplement of potassium chloride, and anti-infection treatment with piperacillin/tazobactam and ciprofloxacin. In the subsequent 2 days, he recovered from coma gradually, but presented clear sign of lung infection (fever, moist rale sound in both sides of lower lung lobes, blood WBC 9.8 × 10^9^/L, NEUT 94.61%, CRP 146 mg/L). Diagnoses of shock, multiple organ dysfunction syndrome (MODS), and lower respiratory tract infection were made. The infection and the respiratory acidosis combined with metabolic alkalosis lasted for nearly the whole 52 day-hospitalization. On the 45th day of hospitalization, a fistula between the esophagus and trachea was found through gastroscopy and further verified by bronchoscopy. It was treated with tracheal stenting 2 days later. However, 5 h after post-tracheal stenting, sudden cardiac arrest occurred, complicated with a loss of spontaneous breathing, then he went into coma (combined with dilated pupils and disappeared light reflex of both sides) again. BP and SpO_2_ could not be measured while his pulse and spontaneous breathing returned 5 min after salvage. BP, SpO_2_, and light reflex of both pupils returned to normal gradually within the subsequent 5 h. However, the patient was still in coma and was discharged after 7 days at his family's request to terminate treatment.

During the hospitalization, the separately or jointed isolated pathogenic microbes for the lung infection included *Acinetobacter calcoaceticus*–*Acinetobacter baumannii* complex (7 times isolated from sputum, 7 TIFS), *Acinetobacter baumannii* (4 TIFS), *Enterobacter cloacae* (3 TIFS), *Pseudomonas aeruginosa* (2 TIFS), *Serratia marcescens* (2 TIFS), molds (2 TIFS), and *E. anophelis* (7 TIFS) (named as 12012 PRCM). *E. anopheles* 12012 PRCM was verified by 16S rRNA gene sequencing (GenBank accession no. KR349263) of the genomic DNA with the forward primer 27F (5′-AGAGTTTGATCCTGGCTCAG-3′) and the reverse primer 1492R (5′-GGTTACCTTGTTACGACTT-3′; Lane, 1991). It was resistant to all the 20 antibiotics in the antibiotic susceptibility tests (AST) panel, including β-lactams (ampicillin, piperacillin, ampicillin-sulbactam, piperacillin-tazobactam, ceftazidime, cefotaxime, cephazolin, cefepime, aztreonam, imipenem, and meropenem), aminoglycosides (amikacin, gentamicin), tetracycline, chloramphenicol, and polymyxin, quinolones (ciprofloxacin, levofloxacin, moxifloxacin), and sulfamethoxazole-trimethoprim. The strain was also resistant to another antibiotic amoxicillin-clavulanate tested. To investigate the resistance mechanisms, extended spectrum β-lactamase (ESBL) detection was performed on the *E. anophelis* 12012 PRCM using BD's PhoenixTM-100 Automated Microbiology System with the NMIC/ID-4 panel (Becton, Dickinson, and Company; Stefaniuk et al., 2003; Liu et al., 2005). The cephalosporin test was positive and the carbapenem-hydrolyzing β-lactamases and carbapenemases (CHBLs) were detected. In addition, we found this strain was co-positive between EDTA (Ethylenediaminetetraacetic acid) and imipenem. These laboratory findings indicated strain 12012 PRCM could produce metal-beta-lactamase, possibly conferring the β-lactamases resistance.

### Case 2

A 47-year-old male with hypotension (BP 95/55 mmHg) was admitted to our hospital on August 21, 2015. He had history of exacerbated abdominal pain and abdominal distension for more than 10 days, shortness of breath for 3 days, complicated with stagnated exhaust and defecation. Before admission, his pressure was maintained by continuous pumping of L-noradrenaline and dopamine. Physical examination found weak breath sounds in both sides of lung lobes and severe subcutaneous edema on whole body. The arterial blood gas analysis displayed acidosis (pH 7.20, pCO_2_ 11.1 mmHg, pO_2_ 99 mmHg, ${\text{HCO}}_{3}^{-}$ −8.6 mmol/L, BE −23.3 mmol/L). He had a low white blood cell count (WBC) (2.35 × 10^9^/L). Blood biochemical test showed several abnormal results (glucose 8.86 mmol/L, BUN 18.93 mmol/L, creatinine 271 μmol/L, GFR 24.20 ml/min, AST 827 U/L, ALT 197 U/L, albumin 30.5 g/L, T-BIL 29.5 μmol/L, D-BIL 12.8 μmol/L, total osmotic pressure 280 mOsm/L). Ultrasound imaging demonstrated liver cirrhosis, echo enhancement in parenchyma of both kidneys and effusion around left renal. Computed tomography scanning revealed inflammatory changes within both sides of the lower lung lobes, severe effusion within bilateral pleural, abdominal, and pelvic cavity, edema within subcutaneous soft tissue of chest and abdominal wall. Diagnoses of shock, MODS, electrolyte imbalances (low sodium, low calcium), diabetes and pulmonary infection were made. Within 16 h after admission, the patient further presented oliguria and aggravated during the 12-day hospitalization. On the 3rd day, hepatic virus infection [with positive HBsAg, anti-HBe antibody, and anti-HBc antibody (IgG)], abnormal blood coagulation [prothrombin time (PT) 16.7 s, partial prothrombin time (APTT) 67.8 s, fibrinogen (FIB) 1.75 g/L, D-dimer 8.32 mg/L, international normalized ratio (INR) 1.43], and thrombocytopenia (84 × 10^9^/L) were diagnosed, with the latter lasting for the remaining hospitalization. The treatment strategies included drainage of bilateral pleural effusion, maintaining blood pressure as previously, improving liver function, daily hemodialysis, management of electrolyte, insulin injection and improving nutrition. The empirical antimicrobial therapy was started with IV piperacillin and tazobactam during the first 3 days of hospitalization, then changed to IV imipenem and cilastatin sodium for 1 day and further combined with vancomycin; the latter lasted for the remaining 8 days of hospitalization. The patient died of sudden cardiac arrest.

By using BD's Phoenix™-100 Automated Microbiology System with the NMIC/ID-4 panel as Case 1, we repeatedly isolate *E. meningoseptica* (on the 7th and 9th day of hospitalization respectively) and *Stenotrophomonas maltophilia* (on the 8th and 9th day of hospitalization respectively) from sputum cultures. Later, *E. meningoseptica* was verified as *E. anophels* (named as N107618, GenBank accession No. KX775952) by 16S rRNA gene sequencing as Case 1. *E. anophelis* N107618 was resistant to 16 out of 20 antibiotics in the same AST panel used to test the *E. anophelis* 12012 PRCM, and was only susceptible to ciprofloxacin, levofloxacin, moxifloxacin, and sulfamethoxazole-trimethoprim.

## Discussion

*E. anophelis* was an opportunistic pathogen but it could result in a high mortality rate (Teo et al., 2013; Lau et al., 2016; https://www.cdc.gov/elizabethkingia/outbreaks/). The number of *E. anophelis* infections was possibly underestimated because *E. anophelis* were often misidentified as *E. meningoseptica* by conventional identification methods (Kämpfer et al., 2011; Teo et al., 2013, 2014; Nicholson et al., 2016). Identification of *E. anophelis* by the matrix assisted laser desorption-ionization time-of-flight (MALDI-TOF) mass spectrometry will be helpful if *E. anophelis* reference is added in the database (Teo et al., 2013; Lau et al., 2016). 16S rRNA gene sequencing should be performed as a routine bacterial identification tool in the medical laboratories without MALDI-TOF mass spectrometry (Teo et al., 2013; Lau et al., 2016).

The high mortality rate of *E. anophelis* infection was partly due to its property of multidrug-resistance. *E. anophelis* N107618 was resistant to 16 out of 20 antibiotics while strain 12012 PRCM was resistant to all the 20 antibiotics in the AST panel, indicating that our two clinical *E. anophelis* isolates were multidrug-resistant. Furthermore, our tests showed that the isolate *E. anophelis* 12012 PRCM produced metallo-beta-lactamase(s). β-lactams resistance has been shown in many clinical important *Elizabethkingia* isolates (Frank et al., 2013; Lau et al., 2015, 2016). Notably, all clinical *E. anophelis* isolates, except for our strain 12012 PRCM, were susceptible to one or more quinolones such as ciprofloxacin or moxifloxacin (Frank et al., 2013; Lau et al., 2015). Therefore, ciprofloxacin or moxifloxacin might be considered as empirical antibiotic therapy of *E. anophelis* infections. For example, Frank et al. reported that a clinical isolate was resistant to all β-lactams tested (amoxicillin, cefotaxime, ceftazidime, cefepime, and imipenem) except for piperacillin and aminoglycosides (amikacin, tobramycin, and gentamicin) while it was susceptible to moxifloxacin (Frank et al., 2013). Similarly, three clinical *E. anophelis* strains were resistant to β-lactams aminoglycosides, tetracycline, and chloramphenicol while they were susceptible to pipercillin, cefoperazone-sulbactam, trimethoprim-sulfamethoxazole, ciprofloxacin, moxifloxacin, and rifampin (Lau et al., 2015). Moreover, the survey of 17 *E. anophelis* bacteremia demonstrated that they were resistant or intermediate-resistant to imipenem, amikacin, gentamicin, and tobramycin, but they were susceptible to ceftazidime, piperacillin, and rifampicin (Lau et al., 2016).

Next generation sequencing (NGS) techniques greatly contribute to our understanding of drug resistance and infectivity of *E. anophelis*. For example, 16 and 19 antibiotic resistance genes were found from the core and accessory genomes of *E. anophelis*, respectively (Teo et al., 2014). The genome of *E. anophelis* NUHP1 contained 14 antibiotic resistant genes that correlated to its multi-drug resistance (Li et al., 2015). Breurec et al. reported that at least 17 antimicrobial resistance genes in *E. anophelis* consisted of metallo-beta-lactamase genes *bla*_blaB_ and *bla*_GOB_ (conferring β-lactams resistance), subclass B1 beta-lactamase gene, efflux systems, conserved chloramphenicol acetyltransferase (*CAT* and *AAC3-I*) gene, a *tetX* gene (encoding for a tetracycline inactivating enzyme) and *LinF* gene (encoding lincosamide resistance protein; Breurec et al., 2016). Genome sequencing analysis showed that *E. anophelis* processed virulence genes encoding cell wall hydrolase A, listeriolysin O (LLO), phospholipase (PlcA), virulence cluster protein B (VclB), and arylsulfatase (Lau et al., 2015; Breurec et al., 2016). These virulence factors possibly contribute to the invading of the host, escape from phagocytosis, surviving in the secondary vacuole of macrophages, and propagating in bloodstreams (Lau et al., 2015; Breurec et al., 2016).

## Conclusion

*E. anophelis* is an emerging opportunistic pathogen. It often leads to a high mortality rate because of its multiple antibiotic resistance. Therefore, *E. anophelis* infections should be considered clinically significant. Investigation of antibiotic resistance mechanisms are warranted.

## Ethics statement

The written informed consents were obtained from the patients or their relatives/guardians. This work was already approved by the Ethics Committee of Quanzhou First Hospital.

## Author contributions

SH and TJ contributed equally to this work. All of authors wrote the papers. SH conducted the test. MW, DM, and SC guided the testing and experiments.

### Conflict of interest statement

The authors declare that the research was conducted in the absence of any commercial or financial relationships that could be construed as a potential conflict of interest.
